# Automatized image processing of bovine blastocysts produced *in vitro* for quantitative variable determination

**DOI:** 10.1038/sdata.2017.192

**Published:** 2017-12-19

**Authors:** José Celso Rocha, Felipe José Passalia, Felipe Delestro Matos, Maria Beatriz Takahashi, Marc Peter Maserati Jr, Mayra Fernanda Alves, Tamie Guibu de Almeida, Bruna Lopes Cardoso, Andrea Cristina Basso, Marcelo Fábio Gouveia Nogueira

**Affiliations:** 1 Universidade Estadual Paulista (Unesp), Faculdade de Ciências e Letras (FCL), Campus de Assis, Laboratório de Matemática Aplicada, Brazil; 2 Institut de Biologie de l'École Normale Supérieure de Paris, Paris, France; 3 In Vitro Brasil SA—Mogi Mirim, Brazil; 4 Universidade Estadual Paulista (Unesp), FCL, Câmpus de Assis, Laboratório de Micromanipulação Embrionária, Brazil

**Keywords:** Embryology, Applied mathematics, Microscopy, Imaging

## Abstract

There is currently no objective, real-time and non-invasive method for evaluating the quality of mammalian embryos. In this study, we processed images of *in vitro* produced bovine blastocysts to obtain a deeper comprehension of the embryonic morphological aspects that are related to the standard evaluation of blastocysts. Information was extracted from 482 digital images of blastocysts. The resulting imaging data were individually evaluated by three experienced embryologists who graded their quality. To avoid evaluation bias, each image was related to the modal value of the evaluations. Automated image processing produced 36 quantitative variables for each image. The images, the modal and individual quality grades, and the variables extracted could potentially be used in the development of artificial intelligence techniques (e.g., evolutionary algorithms and artificial neural networks), multivariate modelling and the study of defined structures of the whole blastocyst.

## Background & Summary

The growing world population is constantly demanding more and better food, which creates an increasing demand for animal protein. Therefore, meat production from cattle is important, and Brazil is a globally important participant in the chain of bovine meat production. Bovine meat production depends on reproduction to maintain and expand the current production. *In vitro* embryo production has gained importance as an assisted reproduction technique in the cattle industry. The large size of the Brazilian cattle herd coupled with the large number of oocytes per *ovum*-pick-up associated with the prevalent Nellore breed (*Bos taurus indicus*), partially explain why Brazil is the main producer worldwide of *in vitro* bovine embryos.

Amongst the limitations of *in vitro* production of embryos is the lack of a consistent and reproducible method to objectively evaluate the morphological quality of embryos. The standard method of quality grade evaluation for bovine species is examination under stereomicroscopy, and is based on three scores (recommended by the International Embryo Technology Society or IETS^1^). The reasons for performing the assessment of embryo quality are to choose what additional techniques could be applied (e.g., cryopreservation), to measure the outcome of distinct systems of *in vitro* production (i.e., looking for those that produce more high-quality embryos), and to predict the success rate (the percentage of pregnancy after the embryo be transferred to a recipient female). Although this standard method is well accepted and possesses some predictive value of the success rate, it is based on the experience of the embryologist and his or her mood and tiredness. A new method that could evaluate the morphology of the embryo with objective endpoints would need the extraction of a maximum number of variables. They could be used to improve processing of the evaluation and improve the whole representation of the embryo.

Image processing reveals information and features that have a scientific purpose, mainly due to computer algorithms’ ability to quantify variables and establish numeric measures. In contrast, visual analysis by humans is based on a comparison between images. In this regard, human vision has difficulties in judging colour or brightness of shapes and features and requires measuring scales or relative size, angle, and positions of several objects to identify their characteristics^2^. Segmentation and texture analysis allows for automatic assessment of important variables from the mathematical and physical background of images, which human vision cannot identify^3^.

In this sense, we linked standard bovine embryo quality evaluation with variables from blastocyst images after their processing and segmentation and automated variable extraction. The images were digitally captured to investigate embryo quality, an important issue in the worldwide chain of *in vitro* production in the commercial market.

Applications of this type of database can be used as an atlas in a tutorial for embryologists inexperienced with bovine blastocyst classification or in specialized *in vitro* fertilization clinics to search for novel, alternative ways to assess the embryo. Computational algorithms can be elaborated, aiming at a better classification of bovine blastocysts, seeking to eliminate human subjectivity, and using a non-invasive technique such as a digital image. Recently, this aim was attained by an automated bovine embryo evaluation based on image processing and artificial intelligence^4^.

## Methods

### *In vitro* production of blastocysts

The bovine blastocysts were produced by the company *In Vitro* Brasil (Mogi Mirim, São Paulo state, Brazil) based on the protocols described by ref. 5. Briefly, ovaries were obtained from cows from a local abattoir. Since the ovaries came from animals slaughtered for commercial purposes, there was no need for a previous approval of the procedure by an ethics committee according to Brazilian law. The *cumulus*-oocyte complexes (COCs) were retrieved by aspiration from antral follicles (ranging from 3 to 8 mm in diameter), and only COCs with two or more *cumulus* cell layers and no signs of oocyte cytoplasm heterogeneity were used. All the media and reagents used for the steps of *in vitro* maturation, fertilization and culture were the same as described in ref. 5. On the 7th day post-insemination of the culture, the structures were evaluated, and only blastocysts (from early to expanded stages and from any quality grade) were submitted for digital image capture. Structures considered as non-blastocysts (i.e., degenerated embryos or embryos without a visible cavity) were not used in that step.

### Embryo image capture and embryologists’ classifications

**Figure 1:**
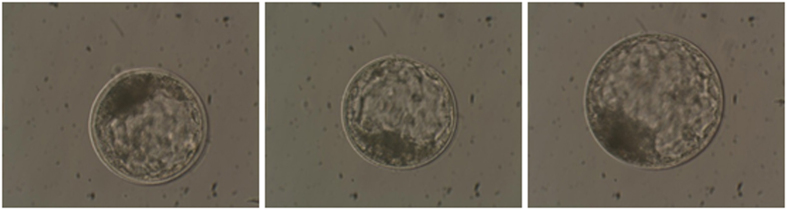
Illustrative image of three different embryos where the major axis of the inner cell mass is positioned perpendicular by its middle section to the focal plane of the microscope.

A database containing 482 images captured of bovine blastocysts was used. The images were obtained using an inverted microscope (Olympus IX71) at 32x magnification and the software Lucam Capture v6.30; images were stored in JPG format in 8-bit colour (RGB) at a resolution of 1,280×1,024 pixels, as described in ref. 4. Each image contained only one embryo, which was approximately centred in the visual field. To capture the image, the position of the blastocyst was standardized such that the plane of focus was in the largest diameter of the embryo and the inner cell mass (ICM) was perpendicular to the focal plane (Fig. 1).

Three experienced embryologists from the company, who were responsible for routine laboratory work, classified each image into three quality grades according to IETS standards^1^. The blastocyst classification (*n*=482) was distributed as follows: 113 images were classified as grade 1 (excellent/good); 175 were classified as grade 2 (fair), and 194 were classified as grade 3 (poor). After the blastocyst classification, the mode of the three evaluations was calculated.

### Image processing

Digital image processing is an essential step to analyse and extract information from the blastocyst images. Digital image processing comprised a series of techniques that have been widely used in solving several problems. This processing ranged from improving photographs to computer vision^6^. An alternative approach to retrieving image features with phase congruency was previously described^7^. Although this technique is dimensionless and useful to avoid standardization of image brightness or contrast, the calculation of phase congruency in an image is computationally expensive^7^. In this way, there are currently some other suitable techniques for image processing. The techniques described in this work are expanded versions of descriptions in our related work^4^.

### Image standardization

Each laboratory applies a different standard to capturing images; therefore, standardizing these methods has become indispensable.

For the segmentation and extraction of information to find similar conditions independently of the images’ original features, all factors must be considered. All of the algorithms were developed using the MATLAB platform^8,9^ and enabled the automated analysis of the images without the need for user intervention.

For standardization, the software consecutively followed the steps of image import, conversion to greyscale, resolution and proportion adjustment, and intensity adjustment, as shown below. This standardization is better explained in our related work^4^.

1. Image import: The image was imported into the MATLAB platform, regardless of image format (BMP or JPG);
2. Conversion to greyscale: Colour information was not used in the interpretation of images and thus all images were converted to 8-bit greyscale. This process also rendered all subsequent steps faster since a decrease of the spectral image size occurs (from the three RGB bands to only one);
3. Resolution and proportion adjustment: In addition to different resolutions, the images had different proportions. An image of 640×480 pixels has a proportion of 0.75, while for an image of 1,280×1,024 pixels, the proportion is 0.8. Therefore, to only resize the images for one of the resolutions would distort the proportions of the other. We choose 640×480 pixels as the default resolution because it is the lowest standard and provides sufficient information for interpretation. Using the lowest resolution also increases the efficiency of the subsequent steps of the algorithm; besides, it does not create minimum resolution limitations for the final software. Therefore, the 1,280×1,024 resolution was adjusted for the ratio of 0.75 (by cutting an upper region of 64 pixels) and then resizing to 640×480 pixels;
4. Intensity adjustment: To minimize the effects of different illuminations, the images were submitted to a histogram adjustment, in which 1% of all information becomes saturated between light and dark pixels. This process also increased the image contrast, which facilitates the subsequent step of segmentation.

### Blastocyst image segmentation

Once standardized, the images underwent a segmentation process, whereby the images were properly isolated from the background and then subjected to information extraction techniques. The steps used in the segmentation algorithm are explained in detail in our related work^4^ and described below.

**Figure 2:**
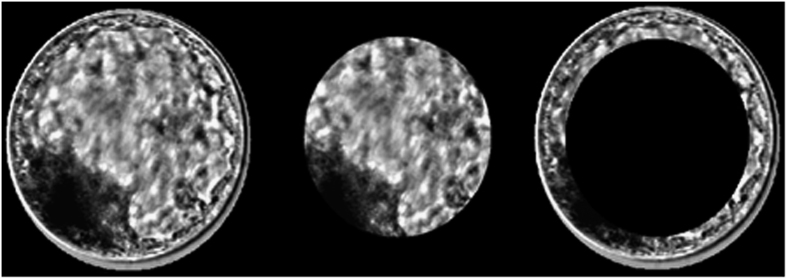
The final version of an isolated image of a bovine blastocyst. On the left, the radius is enlarged by 5 pixels (ER); at the centre, the radius is reduced by 40 pixels (RR); and the right shows the differences between the two radii (TE).

1. Image gradient: Initially, we calculated the magnitude of the image gradient, and the edges were highlighted for subsequent steps. This operation detected edges in all directions, an essential characteristic for the circular shape of the embryo. The greater the intensity of the pixel variation, the greater the resulting magnitude value in the final gradient;
2. Binary image: In this step, we calculated the binary image and selected a value of 128 as the intensity threshold (because it was an 8-bit image with 255 as the maximum intensity value for each pixel);
3. Circular Hough Transform: In this work, we used the circle Hough transform (CHT), which has become widespread in circle detection processes in digital imaging^10^. Once the binary image was obtained, the Hough transform detects the embryo circumference by mapping the image and thus provides an isolated embryo background image. The algorithm performed the detection of circles in two stages: in the first stage, it searches by circles of radius between 100 and 150 pixels; then, it searches radii between 150 and 250 pixels for greater accuracy. These values were obtained by algorithm verification using the entire database. Therefore, both initial blastocysts (smaller) and expanded blastocysts (larger) can be correctly detected. At the end of both searches, in each image, the detected circle's metrics are compared and the largest radius is used after the best circle is detected;
4. Blastocyst isolation: After circumference detection identified the blastocyst, we used this next step to isolate it. Three versions of the blastocyst image were generated. In the first, the radius of the circumference is increased by 5 pixels to make sure the *zona pellucida* is included (called ER); in the second, the radius was reduced by 40 pixels in order to discard the trophectoderm, selecting the inner cell mass (ICM) and blastocoel for analysis only (called RR); and in the third version, we obtained the difference between ER and RR. Therefore, only the trophectoderm region was isolated in the image (called TE; Fig. 2). The pixel values, which determined the expansion (ER) and the contraction (RR) of the blastocysts images, were obtained by assessment of the image database.

### Texture analysis

The image texture was defined by repeated random regular patterns in a region of the image that provided information on the surface structure^11^. This variable is considered an important characteristic that is used to identify the regions of interest in an image^12^.

Among the statistical methods used to analyse the textures in images, the grey level co-occurrence matrix (GLCM) is considered to be among the most efficient^11,13,14^. GLCM describes the spatial distribution of the intensity values of the pixels by considering a determined distance and angle, which makes it possible to recognize and classify textures. Each matrix is the probability of two neighbouring pixels (one with intensity *i*and another with intensity *j*) at a determined distance *d* and angle *θ*, which forms *P*=(*i*, *j*, *d*, *θ*). For the calculation of GLCM, the image intensity was adjusted to have only 8 shades of grey.

### Watershed transform

The image segmentation step has a substantial influence on the independent interpretation of different regions. Two main approaches can be used for segmentation: detecting edges, which delimits a region, or searches for regions that have similar pixel intensities. The watershed methodology searches the targeted image using the second strategy^15^. The watershed transform proposes a morphological approach to the image segmentation problem through its interpretation of pixel intensities as surfaces, in which the grey levels of each pixel determine the height of a given region. Based on this concept, drainage basins, which are defined by regions of local minima and their domain regions, can be identified^16^.

**Figure 3:**
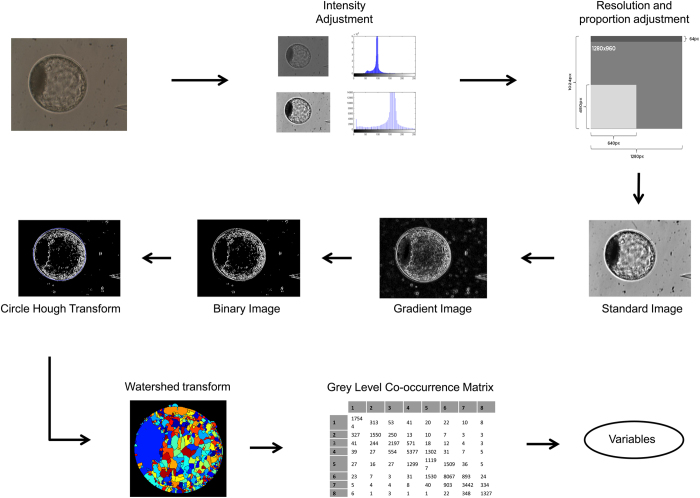
Flowchart of bovine blastocyst image processing.

All the steps involved in this process, including its image processing, standardization, segmentation, and analysis until achieving the quantitative variables calculations, are described in Fig. 3. In this figure, each region identified in the blastocyst segmentation by the watershed transform was, for visualization purposes, assigned a random colour. The ICM has the largest area after segmentation, probably because it is a relatively homogeneous dark region. Therefore, the largest segmented area was used as an ICM mask in the variable extraction steps.

### Description of the chosen variables

After image standardization and segmentation, 36 variables were extracted, which are described below. This is a detailed description from our related work^4^.

The notation ER was used to refer to the blastocyst image version with the radius expanded by 5 pixels, RR to refer to the image generated by radius reduction by 40 pixels and TE to refer the difference between the two radii.

#### 1) Contrast RR

GLCM determined variable. Contrast is the measurement of the intensity difference between a pixel and its neighbours across the entire image with a constant image of zero contrast. This is calculated by equation (1):

(1)∑i,j|i−j|2p(i,j)

where *i*, *j* and *p* are defined in the texture analysis item.

#### 2) Correlation RR

Demonstrates the correlation between the image pixels in determined neighbours across the entire image. A value of −1 or 1 shows a perfectly correlated image, negative or positive, respectively. This value is determined by equation (2):

(2)∑i,j(i−µi)(j−µj)p(i,j)σiσj

#### 3) Energy RR

Sum square of the GLCM elements. An energy value equals to 1 corresponds to a constant image. This value is represented by equation (3):

(3)∑i,jp(i,j)2

#### 4) Homogeneity RR

Proximity measurement of the distribution of GLCM elements with the GLCM diagonal. The homogeneity value is 1 for a diagonal GLCM. This value is represented by equation (4):

(4)∑i,jp(i,j)1+|i+j|

#### 5) Contrast TE, 6) Correlation TE, 7) Energy TE and 8) Homogeneity TE

These variables follow the same principle of variables 1, 2, 3 and 4, respectively. However, this is used as the basis for the TE region instead of RR for the GLCM calculations.

#### 9) DC1

Hough transform for the dark circle detection with a radius between 4 and 8 pixels. The result is the quantity of found circles, with a 0.935 sensibility index (the standard value used in the accumulative matrix of the transform).

#### 10) Mean DC1

Mean of luminous intensity of all circles detected in DC1. The circle centres and their mean radii are used in the construction of a binary mask, which is applied in the original image of the segmented blastocyst and allows for the isolation of circles only. Then, the mean of the luminous intensity in the isolated regions is calculated.

#### 11) LC1 and 12) Mean LC1

These variables follow the same standard of the variables DC1 and Mean DC1, respectively. However, in the variables LC1 and Mean LC1, light circles are detected.

#### 13) DC2 and 14) Mean DC2

These variables follow the same standard established in the variables DC1 and Mean DC1, respectively. However, circles of radii of 9 to 15 pixels with 0.94 sensibility are detected.

#### 15) LC2 and 16) Mean LC2

These variables follow the same standard of the variables DC2 and mean DC2, respectively. However, in the variables LC2 and mean LC2, light circles are detected.

#### 17) Radius ER

Calculated as half of the width of image ER.

#### 18) Sum ER

A binary ER image is calculated using the Otsu algorithm; see ref. 17 for threshold detection. Next, the sum of all values from the binary image is calculated. Finally, this value is divided by the total area of the blastocyst.

#### 19) Mean grey ER

Mean grey intensity of the pixels in ER.

#### 20) Deviation RR

The standard deviation of the grey intensity of the pixels in RR, which is calculated by the equation (5), where *x* is the image intensity vector and *n* the number of vector elements.

(5)StdDeviation=(1n−1∑i=1n(xi−x¯)2)12

where $\bar{x}=\frac{1}{n}\sum_{i=1}^{n}x_{i}$

#### 21) Mean grey RR

Calculated by the same way as mean grey ER but using the region of RR.

#### 22) Mode value RR

The mode value of RR, i.e., the value of luminous intensity occurring most frequently in RR.

#### 23) Dark RR

Initially, pixels with luminous intensity less than or equal to 25, which is 10% of the limit allowed (remembering that as pixels are 8-bit values, the luminous intensity varies between 0 and 255). Then, this value is divided by the total area of the embryo.

#### 24) Mean Count RR

All the pixels with luminous intensity between 10 pixels below and 10 pixels above than the mean intensity were counted. Then, this value is divided by the total area of the embryo.

#### 25) Bright RR

Same as dark RR but using pixels with an intensity higher or equal to 230 (lightest 10% of the image).

#### 26) Deviation TE, 27) Mean grey TE, 28) Mode value TE, 29) Dark TE, 30) Mean Count TE, and 31) Bright TE

These variables follow the same standard of variables 20 to 25, respectively. However, the TE image is used as a basis for calculation.

#### 32) WSN

The total number of regions found by the watershed transform.

#### 33) Area ICM

Area of the largest region found by the watershed transform, corresponding approximately to the ICM area.

#### 34) Convex ICM

Area of the smallest convex polygon, which has the largest detected region by the watershed transform. Thereby, a reliable representation of the ICM real area is intended.

#### 35) Eccen ICM

The eccentricity of the largest region detected by the watershed transform. A value of zero would indicate a perfectly circular area and a value of 1 a line segment. Considering an ellipse that has the approximate shape of the largest region detected by the watershed transform, Eccen ICM is calculated as being the relationship between the ellipse focus distance and the length of its largest axis.

#### 36) Mean ICM

Luminous intensity average value of the largest region detected by the watershed transform.

From all variables obtained with the process described, there are some with a strong correlation between the quantitative variable and its biological meaning (based on the embryo image characteristics; Table 1). However, in some cases, the variable is purely extracted from a mathematical process and it is not directly correlated with any biological characteristic of the image (Table 1 and ref. 4).

### Code availability

The homemade software used in image processing was developed in the MATLAB platform including functions from Image Processing and Image Acquisition toolboxes (MATLAB 2017a, The MathWorks Inc., Natick, MA). The image processing code developed on MATLAB is available at Figshare as files with.m extensions together with the present dataset (Data Citation 1). The version used for programming was R2017a, but the code is also compatible with earlier released versions (e.g., R2012).

### Software versions

The authors suggest the use of recent MATLAB versions for image processing due to toolbox function updates in the newest versions. The releases after version 2014b offer a new graphic system and other functions that improved image-processing techniques (https://www.mathworks.com/help/matlab/graphics-changes-in-r2014b.html).

Other software commonly used in image processing is ImageJ (ImageJ, U. S. National Institutes of Health, Bethesda, Maryland, USA), an open source program that has similar image processing functionalities that were used to analyse the embryo images. The software Lucam Capture v6.30 was used to capture the image from an inverted microscope (Olympus IX71) at 32x magnification.

## Data Records

The data record associated with this paper is available at the Figshare repository (Data Citation 1). All the 482 blastocyst images described in the methodology step ‘Embryo image capture and embryologists’ classifications’ are identified by a title beginning with Bovine blastocyst image—blq, followed by a number *n*, which ranges from 1 to 482 in *.jpeg. All images are also provided as a zip archive (‘Blastocyst images.zip’).

The variables extracted from the images, which were determined by ‘Description of the chosen variables’, are described in the file named ‘Quantitative variables and classification of bovine blastocysts’ in *.xls format (available at the Figshare repository). Each row describes a bovine blastocyst image (identified by the ID previously described), and columns D to AM describe the quantitative variables. The columns AN, AO, and AP are related to the embryologist's classification and the AQ represents their mode.

## Technical Validation

Three highly experienced embryologists conducted the experiments and embryo production, and performed the blastocyst evaluation according to the IETS grading system. The Kappa index among the embryologists was 0.572 (482 images, *P*<0.001), which shows that there was a moderate agreement among the evaluations. The inverted microscope (Olympus IX71 at 32x magnification) used to take the blastocyst images that compose the data records from this paper were maintained under technical and professional conditions and calibration. The images were saved by well-established software (Lucam Capture v6.30) with proper image resolution to guarantee quality and high standard representations of the blastocysts.

The image processing techniques used in this work are approved worldwide and used by the scientific community, as well as analysed by peer-reviewed journals and image processing books^2,18,19^.

## Usage Notes

Each image contained only a single embryo, which was approximately centred in the visual field. To capture the image, the position of the blastocyst was standardized such that the focal plane was positioned at the largest diameter of the embryo and the inner cell mass (ICM) was perpendicular in its centre (i.e., the widest segment of the ICM that was also perpendicular to the focal plane). In a pilot experiment, we observed that when the images were captured with the blastocyst positioned 90° rotated (i.e., the major plane of the ICM parallel to the focal plane) there was no benefit in terms of quantitative variables extracted from the image. In fact, in this attempt, when the ICM’s major plane was in focus, the trophectoderm (TE) was not. To retrieve some information from the TE, the focal plane was placed out of the major plane of the ICM and the quality of the information (quantitative variable) was decreased.

To obtain the 36 variables that characterize the bovine blastocyst, it is necessary to use the main code entitled ‘gerainputs.m’, which is available at the Figshare, in conjunction with the jpg. embryo image file.

## Additional information

**How to cite this article:** Rocha, J. C. *et al.* Automatized image processing of bovine blastocysts produced *in vitro* for quantitative variable determination. *Sci. Data* 4:170192 doi: 10.1038/sdata.2017.192 (2017).

**Publisher’s note:** Springer Nature remains neutral with regard to jurisdictional claims in published maps and institutional affiliations.

## Supplementary Material



## Figures and Tables

**Table 1:** Biological and mathematical descriptions of the 36 quantitative variables from image processing.

**Variable**	**Image processing step**	**Description**
Contrast RR, Energy RR, Homogeneity RR, Contrast TE, Energy TE and Homogeneity TE	GLCM	The statistical method used to analyse the texture of the image and considered to be one of the most efficient. Biologically, they represent cellular homogeneity within the embryo image.
Correlation RR, Correlation TE and Radius ER	Mathematical operations	Mathematical operations. Additionally, they represent the homogeneity of the embryonic cells in the image.
DC1, Mean DC1, LC1, Mean LC1, DC2, Mean DC2, LC2 and Mean LC2	Hough Transform	It defines the circles around intact embryos in digital imaging. The variables identify rounded structures such as extruded non-degenerated blastomeres.
Sum ER	Otsu algorithm	Used in binary image calculation. It highlights the biological aspect of cellular edges with more intense contrast.
Mean grey ER, Mean grey RR, Mean grey TE, Mode value RR, Mode value TE, Deviation RR and Deviation TE	Grey intensity	Pixel grey intensity of three versions of the blastocyst image: expanded radius by 5 pixels (ER); reduced radius by 40 pixels (RR); and the difference between the two radii (TE). They represent, respectively, the average grey intensity of the entire blastocyst (ER), the embryo without the trophectoderm (RR) and primarily the trophectoderm (TE).
Mean Count RR, Bright RR, Mean Count TE, Dark RR, Bright TE, Dark TE	Luminosity intensity	Variables defining pixels with various luminous intensities. They do not correlate with any biological aspect of the embryo.
WSN, Area ICM, Convex ICM, Eccen ICM and Mean ICM	Watershed Transform	A morphological approach to overcome the segmentation issue. It understands the pixel intensities as surfaces where light pixels are high and dark pixels are low. It uses variables that are related to the visual differences and similarities of each region from the embryo.
